# The CORE Group Partners Project in North East Nigeria: Community Engagement Strategies to Combat Skepticism and Build Trust for Vaccine Acceptance

**DOI:** 10.4269/ajtmh.19-0143

**Published:** 2019-10

**Authors:** Samuel Usman, Lydia Bologna, Katherine V. Stamidis

**Affiliations:** 1 CORE Group Partners Project/Nigeria, Abuja, Nigeria;; 2 CORE Group Polio Project, Washington, District of Columbia

## Abstract

In North East Nigeria, anti-immunization rumors and sentiments have negatively impacted the country’s polio eradication efforts. Since 2014, the CORE Group Partners Project (CGPP) has leveraged local-level strategies to help change prevailing attitudes and behaviors by improving immunization acceptability in some of the most difficult settlements in Nigeria’s states at highest risk for polio. The CGPP’s communication model in Nigeria, in part, emphasizes the need to counter suspicion and address myths and misunderstandings by convening community dialogs and compound meetings, both of which serve as safe spaces for open discussion primarily aimed at addressing non-compliance. In the communities in Kaduna, Katsina, Kano, Borno, and Yobe states located in the CGPP implementation areas, there has been a consistent reduction in the number of missed children and consistent improvement in polio immunization uptake, providing evidence of the effectiveness of the CGPP communication model. The last case of wild poliovirus in Nigeria was detected in August 2016. Since Nigeria has gone more than 3 years without a case of wild poliovirus, the CGPP communication model promises to remain highly relevant in sustaining the community’s awareness about immunizations that will be required to keep the population coverage of polio immunization high and, by extension, the herd immunity required to maintain zero transmission of poliovirus in Nigeria. This article describes the various strategies used to address noncompliance and provides examples of community engagement in Yobe state, which is one of the project’s largest implementation areas.

## INTRODUCTION

As the world moves closer to polio eradication, eliminating the poliovirus from Nigeria, Afghanistan, and Pakistan is a substantial challenge. The fight against polio in Nigeria has been marked by strong achievements and tested by significant setbacks. By 2015, Nigeria was on track to be polio free. However, this achievement was short lived, as two cases of indigenous wild poliovirus (WPV) were identified in August 2016. Unreachable and unimmunized children, unreliable coverage data, inadequate surveillance, vaccine myths and hesitancy, and violence from insurgencies all contributed to this failure.^1–3^ These challenges continue to plague underserved and fragile areas of the country, particularly the North East States, which have also been affected by ongoing circulating vaccine-derived poliovirus type 2 (cVDPV2) outbreaks† beginning in 2018.

Perhaps, the greatest overarching challenge has been produced by Boko Haram, a fundamentalist Islamic group that has launched violent attacks in Northern Nigeria and in the neighboring countries of the Lake Chad basin region—Niger, Chad, and Cameroon. From June 2011 through June 2018, the Nigeria Security Tracker documented 2,021 incidents involving Boko Haram, in which 37,530 people were killed, nearly double the conventionally cited estimate of 20,000.^4^ Over the same period, the Armed Conflict Location and Event Data Project, an independent non-governmental organization based at the University of Sussex, identified 3,346 incidents, in which 34,261 people were killed.^4^

The consequences for the polio eradication initiative have been near-catastrophic. Boko Haram has targeted and killed polio workers, disrupted polio campaigns, and damaged or destroyed health facilities. In May 2013, the Nigerian President Goodluck Jonathan declared a state of emergency in Yobe, Borno, and Adamawa states because of the activities of Boko Haram.^5,6^ Moreover, Northern Nigeria has historically experienced large-scale rejection of polio immunization initiatives because of widespread rumors, myths, and religious beliefs. The 2003–2004 polio immunization boycott in Northern Nigeria fueled further outbreaks of polio. In 2017, an outbreak of monkeypox was rumored to have been linked to the polio immunization program and led to resistance in some communities. In some areas of Northern Nigeria (as well as in Cameroon), some people have erroneously come to believe the polio immunization can produce sterility in women.^7^

## THE CGPP IN NIGERIA

The CGPP is a multi-country, multi-partner initiative funded by the United States Agency for International Development to support Nigeria’s polio eradication initiative and routine immunization efforts in five states located in Northern Nigeria. These states are considered at high risk for polio transmission because of low routine immunization coverage, gaps in surveillance, and barriers related to access and security. A history of the global CORE Group Polio Project is available elsewhere in this series.^8^ The CGPP in Nigeria substitutes the term “Partners” for “Polio” in its name because of the sensitivity of the term “polio” in the northern region of the country; the project in Nigeria is also referred to as CGPP as well. In Nigeria, the CGPP encountered several challenges at the onset of implementing the program: low awareness and low uptake of polio and routine immunizations; male heads of households disallowing mothers and caregivers from presenting their children for immunization; poor community participation in immunization activities (resulting in no volunteers being recruited from the communities to support immunization activities); myths and misconceptions about the polio vaccine and unavailability of reliable immunization data at the local government area (LGA) level.

To address the many challenges to immunization uptake in the North, the CGPP began implementing strong community-based engagement strategies to counter rumors, myths, and misconceptions that hindered polio immunization acceptance in the North East. Key approaches used by CGPP included community participatory dialogues, neighborhood meetings, training of male peer educators, and involvement of Muslim religious leaders to encourage fathers to support polio immunization acceptance. Engaging the community led to the acceptance of polio immunization among communities previously opposed to all immunizations. A separate article in this series^9^ analyzes the role of the volunteer community mobilizers (VCMs) in Nigeria, who made these transformations possible.

The CGPP’s contribution to the polio eradication initiative in Nigeria began in January 2014. Initially, the CGPP’s mandate called for it to focus program activities in North West Nigeria. Plans quickly changed once the National Polio Emergency Operations Center (referred to as the NEOC)‡ directed the CGPP to “follow the virus.§” Hence, the CGPP concentrated its efforts in the states of Borno and Yobe—in the North East, where the most recent cases of polio caused by WPV had been detected.

When the CGPP launched its work in North East Nigeria, an assessment by the World Bank showed that more than 40% of the health facilities had been either damaged or destroyed, greatly limiting access to routine immunization services and increasing the risk of disease outbreaks.^10^ The North East states were plagued by high levels of extreme and recurring violence, making it difficult to reach children with polio immunization and making the states particularly vulnerable to transmission of poliovirus.^10^

### A situational analysis in Yobe state.

At the time the CGPP began its work in 2014 in Yobe state, the population coverage of the third dose of oral polio vaccine (OPV3) was 27.2%, Diphtheria, Pertussis, and Tetanus (DPT3) coverage was 11.0%, and the coverage of the third dose of pentavalent vaccine‖ was 7.8%, whereas the national coverage levels were considerably higher.^11,12^ These findings indicate that at the time, there was a low awareness about and limited access to routine immunization, including polio immunization, as well as a health system that was ill-equipped to meet even the basic health needs of particularly vulnerable children. Immunization coverage has always been low in the North East Nigeria, and Yobe and Borno states have been the most affected by the Boko Haram insurgency. Yobe state was beleaguered with rampant violence, hard-to-reach populations, a dilapidated health system and, not surprisingly, low immunization coverage. The CGPP initiated full-fledged interventions in 2014 to reach as many children as possible with polio immunization in Yobe state.

### Female volunteer community mobilizers and male engagement.

Volunteer community mobilizers are the foundation of the CGPP efforts in Nigeria. All VCMs are women, and they are selected by and from their local communities to serve as volunteers. They are trained and supervised by the CGPP and its partners. Volunteer community mobilizers are assigned households in their community and responsible for making frequent visits to speak with caregivers about polio immunization and routine immunization, aiming to dispel misconceptions. Volunteer community mobilizers in each settlement of the CGPP implementation areas regularly record child immunization, track pregnant women, and register births in their settlements to ensure that as many newborns as possible receive their birth immunization against polio (OPV0) as well as other routine immunization in a timely manner. They establish personal relationships with the families they serve, building trust and becoming valued sources of information. During immunization campaigns, VCMs mobilize the caregivers in their communities to immunize their children.

The VCMs go house to house to support families, resolve issues of non-compliance when they can, and record cases where noncompliance could not be resolved. Volunteer community mobilizers frequently attend naming ceremonies (*suna*) and other religious gatherings where they administer OPV0 to newborns. The CGPP has continued to build the capacity of VCMs to provide more information and support to their communities. They have been trained in breastfeeding, nutrition, and child health. In addition, they are equipped to refer families for other services when indicated.

Volunteer community mobilizers are supervised by volunteer ward supervisors and LGA coordinators who provide supportive supervision, on-the-job training, and mentorship to improve the accuracy and efficiency of activities and reporting. Data are reported weekly, enabling the CGPP to stay abreast of any changes or needs in the communities located in its implementation areas. Positions initially reserved for men such as the volunteer ward supervisors and community informants are now frequently filled by females, but the importance of male involvement has not been disregarded.

The CGPP has recognized the critical role of men as decision makers in the communities in its implementation areas and has sought to find ways to engage them—especially those who are gatekeepers (e.g., traditional, religious, and educational leaders)—in unique strategies. One successful example of male engagement is the Iftar¶ Intervention. The intervention was designed to resolve polio immunization rejection through the engagement of male heads of households following evening prayers held during Ramadan.# Male religious leaders and other male gatekeepers were trained to deliver supportive immunization messages.

Before the start of these efforts, VCMs create a list of children who did not receive polio immunization because of rejection by their caregiver along with the name of the mosque their fathers were likely to attend. Consultations with imams†† yielded an estimate of the number of male worshippers likely to attend services on the days of implementation. On the day of the intervention, messages about polio immunization and the importance of immunizations in general were given by imams and LGA supervisors. Male heads of household were given opportunities to ask questions, and misconceptions and myths were addressed by the team. The fathers were encouraged to commit to immunization and bring their children for immunization. The following day, vaccinators were available after prayer services to immunize children. Overall, 97% of children from non-compliant households (2,518/2,606) were immunized in 2018.

### Resolving noncompliance in Yobe state.

The 2018 National Nutrition and Health Survey^11^ revealed that 57.2% of children 12–23 months of age had received their third dose of pentavalent vaccine in the whole of Nigeria. Although this indicates an improvement from 48.8% in 2015, the data were more troubling in the conflict-affected Yobe state. The survey showed that 48.4% of children aged 12–23 months had received the third dose of the pentavalent vaccine. Although this represented major progress from the 2014 coverage level of 7.8%, it was still almost 10 percentage points below the national average.

In the LGA of Karasuwa in Yobe state, the CGPP had been mobilizing communities for immunization acceptance through interpersonal communication. These efforts proved successful. The percentage of zero dose children (children who had never received a dose of polio vaccine) decreased from 1.0% to 0.2% in 2018, the lowest rate ever recorded. In addition, there were no areas in which acute flaccid paralysis (AFP) was not detected (although none of the cases of AFP turned out to be due to polio), meaning that cases of AFP were being detected through the surveillance system and that the surveillance system was quite sensitive.‡‡ This latter accomplishment can be credited at least in part to the consistent effort of the CGPP-supported community informants,§§ who serve as community-based surveillance agents to identify children younger than 15 years with signs of AFP. The CGOO VCEMS are also trained‖‖ to report cases of suspected AFP and report more cases of AFP than do community informants. This could be the result of the greater frequency of household visits [i.e., more contacts] by VCMs). In addition, CGPP VCMs have been able to reduce noncompliance in various communities in Yobe and in the other four states through their skill in tracking parents who default from routine immunization and guiding them for follow-up at the health facility. Some examples of the well-established defaulter-tracking skills of CGPP VCMs are described in the following paragraphs.

After every routine immunization session, the health provider extracts and shares with the CGPP VCM attached to the health facility a list of children who had defaulted. The VCM tracks down these defaulters to ensure that they are reconnected to the health facility to continue receiving their routine immunizations. In Yobe state, for example, one of the CGPP VCMs from Bursari LGA (specifically the Adamu Kellumi settlement in Dapchi ward) was assigned to track defaulters from her settlement. Within 2 weeks, she was able to reconnect with all 21 caregivers who had a child younger than 1 year and who had defaulted. She was able to get all of these children caught up on their immunization schedule.

In addition, the s*una* vaccination (the polio vaccination given during the mandatory naming ceremony that occurs 7 days after birth) has increased the number of children being immunized with the birth dose of OPV. Pregnant women are being tracked and monitored so that all deliveries can be documented and the VCM can vaccinate the baby during naming ceremony on the seventh day.

In April 2018, the CGPP joined the government and other polio eradication partners to launch the African Vaccination Week under the umbrella theme of “Vaccines work. Do your part!” Organized and led by the WHO, the annual event promotes increased awareness of the rights and needs of women and children to be protected from vaccine-preventable diseases through vaccination. The CGPP worked with the government and partners such as the United Nations Children’s Fund to pioneer the commemoration of African Vaccine Week at the LGA level as a means of making the local government more aware and accountable on immunization issues. The CGPP supported key components of African Vaccine Week such as message development for use in information, education, and communication materials, and it also generated awareness and understanding through advocacy and social mobilization activities.

Several households in Anoma settlement (part of Karasuwa LGA in Yobe state) reacted to rumors that vaccines would sterilize their children and that they contained “impurities” that would taint Muslims, making them “unholy.” There had always been anti-vaccine rumors, which affected the uptake of immunization services. During this special week-long event, five households with nine children in the Anoma community were unwilling to accept the vaccine. After learning of the community’s rejection, the LGA government immunization team assigned the local CGPP team to meet the community members and address their concerns. Vaccine opposition had been a recurring issue in the LGA, prompting the NEOC to call upon the CGPP to change resisters into accepters.

The CGPP held compound (neighborhood) meetings, community dialogue, and face-to-face interactions using CGPP information, education, and communication materials. The CGPP was given permission to visit households. The CGPP team conducted a dialog with all the caregivers of the settlement. One reason given by the caregivers for rejecting polio vaccine was the following: “*Duk lokacin da yaran mu ba su da lafiya*, *ba’a bamu Magani a Asibiti, ama kuna so ku ba wa yara masu lafiya alluran rigakafi bayan ba su bukatan shi*.” (“When we or our children get sick, we can’t get medication at the hospital, but you want to help the children when they don’t need any help by giving them vaccines.”)

The CGPP team helped the mothers to understand the importance of vaccines by emphasizing that the free immunizations offered immunity from diseases that could sicken and possibly cause death in their children. After 90 minutes of lively discourse and engagement by the CGPP team,¶¶ the caregivers grew to understand the benefits of the polio vaccine.## The caregivers thereafter made a commitment to take their children for routine immunizations at a health facility 5 km away. The effect of word of mouth was instant: the very next day, 58 of 102 children from 35 households obtained their polio immunization.

Just 1 month later (in March 2018), the CGPP worked to resolve another case of non-compliance in Yobe state. The Tsohuwar Kasuwa settlement in Gadaka/Shembire Ward has among the highest numbers of noncompliant households, according to the Ward review meetings that take place between immunization campaigns. To combat cases of immunization resistance during house-to-house campaigns in Fika LGA, the CGPP organized immunization teams to meet the members of resistant households to discuss immunizations and to listen to the concerns of non-compliant community members. The team successfully convinced seven noncompliant households to immunize all of the 28 children in these households. Efforts such as these led to increasing the number of immunized children and decreasing the number of noncompliant households at the local level.

## THE ACHIEVEMENTS OF THE CORE GROUP POLIO PROJECT IN NORTH EAST NIGERIA

In 2014, the CGPP in Nigeria began with 647 VCMs and 106 volunteer ward supervisors across 23 LGAs in the five high-risk states of Kaduna, Kano, Katsina, Borno, and Yobe. These states had high rates of immunization rejection and noncompliance along with low rates of polio and routine immunization uptake. By September 30, 2018, the CGPP had grown to include 2,205 VCMs and 264 volunteer ward supervisors in 32 LGAs in the same five states, covering 598,781 households, an increase from 355,197 households in 2017. This expansion in coverage translates to more pregnant women identified, tracked, and referred for antenatal care along with higher levels of newborn tracking and immunization with OPV birth dose and routine immunizations. On average, 835 newborns and 7,165 children were tracked and immunized with OPV each month.

**Figure 1.**
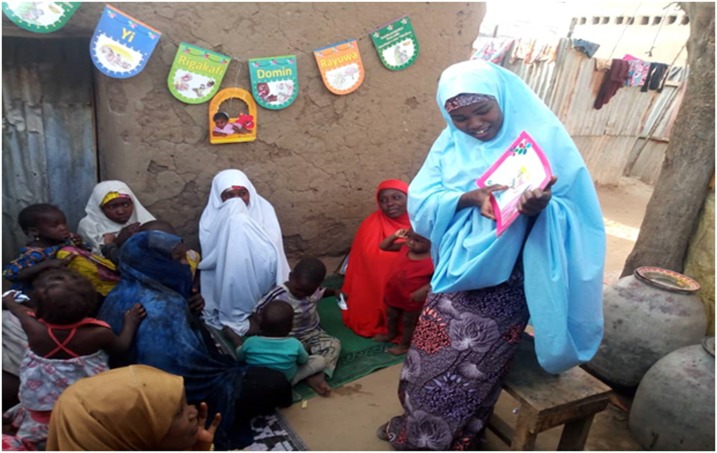
In Yobe state, VCM Yagana Inuwa conducts a compound meeting in the Daki Tara settlement of Sabon Gari Kanuri Ward in Nguru local government area. The volunteer explains the importance of routine immunization and key household and hygiene practices. Photo credit: Ramatu Musa Idiriss, the CGPP Volunteer Ward Supervisor. This figure appears in color at www.ajtmh.org.

**Figure 2.**
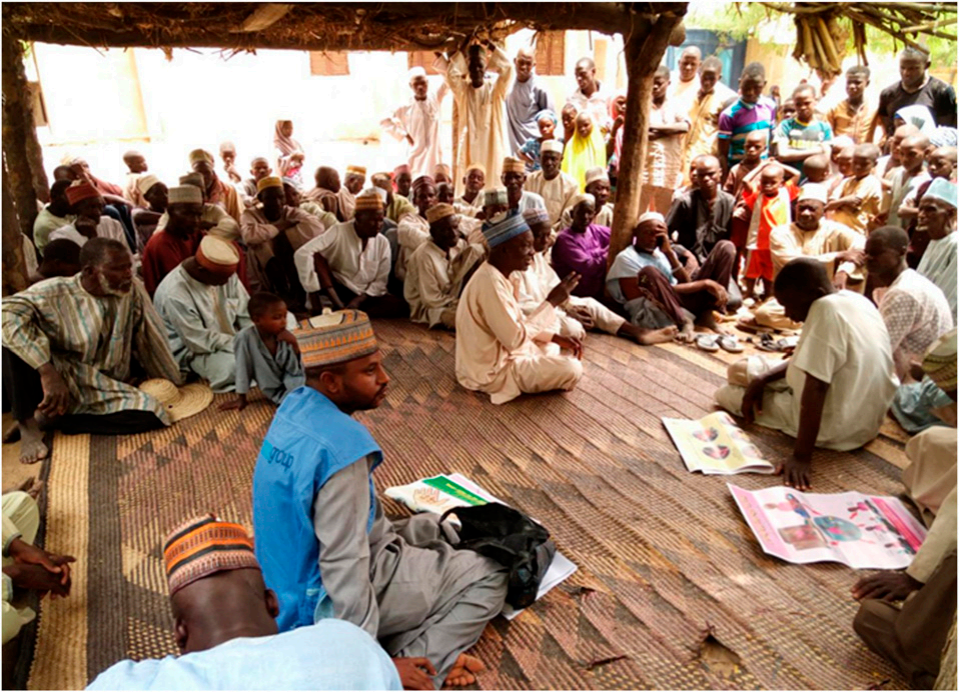
In Yobe state, Bulatura ward, the men of the Sunomari settlement convene for a community dialogue led by the coordinator for Yusufari local government area. Photo credit: Ramatu Musa Idiriss, CGPP Volunteer Ward Supervisor. This figure appears in color at www.ajtmh.org.

The CGPP VCMs have continued to track the immunization status of children, newborns, and pregnant women. They have developed strong knowledge and interpersonal communication skills, enabling them to resolve most issues of immunization noncompliance. Figure 1 captures a compound meeting and Figure 2 a community dialogue in Yobe state.

The impact of these efforts is visible through reports provided by the VCMs in the CGPP implementation areas. Coverage of polio immunization and routine immunization has climbed steeply among children and families in CGPP implementation areas.^13^ According to the CGPP evaluation reports, OPV0 coverage increased from 54.8% in 2014 to 99.0% in 2018 and OPV3 coverage among children aged 12–23 months increased from 47.2% in 2014 to 62.3% in 2017 and 88.4% in 2018.^13,14^ Routine immunization coverage has also increased during CGPP implementation: the percentage of fully immunized children rose from 33.0% at the inception of the CGPP in 2014 to 57.0% at the end of 2017 and 67.0% in 2018.^13,14^

In addition, extensive community mobilization during campaigns has contributed to a marked increase in the number of children younger than 5 years reached during each supplemental immunization activity, increasing from 355,148 children in 2014 to 837,454 in 2018. The percentage of missed children during the supplemental immunization activities based on reports from VCMs has also gradually reduced in the CGPP areas from 4.5% in 2014 to 0.8% in 2018.^14^ The CGPP has also made significant contributions to the polio surveillance network in the five states where it is working, engaging 792 community informants in addition to the VCMs. By the end of 2018, despite not having a presence in all settlements of the LGAs where the CGPP was working, 18.3% (41 cases) of the total 224 identified AFP cases were reported by CGPP-supported VCMs and community informants.

Yobe state is currently one of the largest CGPP implementation areas. The CGPP works in 10 LGAs there, using 780 VCMs and 78 volunteer ward supervisors. According to the CGPP’s records, the VCMs visit an average of 59,000 households there each week (an average of 76 homes visited each week by each VCM). The VCMs also attend naming ceremonies to administer OPV0. At least 3,750 children younger than 5 years are immunized weekly against polio in the state across the 10 LGAs where the CGPP is active.

## DISCUSSION

The findings presented here suggest that the efforts of the CORE Group Polio Partners (CGPP) VCMs have contributed to improved immunization coverage in areas of CGPP work, especially in North East States such as Yobe. In most areas of Nigeria’s North East, the level of insecurity is such that only CGPP and UNICEF VCMs are presently mobilizing the communities. A companion article in this series^9^ describes further the contributions of VCMs along with the non-governmental and civil society organizations with which they worked that contributed to these achievements.

Nigeria has now gone almost 3 years without detection of a case of WPV and is on track to possibly achieve a WPV-free-status in September 2019. However, the challenge of cVDPV remains a major hurdle to achieving complete polio eradication, and the cVDPV challenge highlights serious gaps in routine immunization that is mainly due to low coverage for inactivated polio vaccine. The CGPP and other Global Polio Eradication Initiative (GPEI) partners (especially UNICEF) have contributed to this important milestone of near attainment of WPV-free status in Nigeria by raising awareness regarding the importance of polio immunization in previously non-compliant communities. The CGPP currently works in five of the 11 states in Northern Nigeria that are at high risk for continued polio transmission. It has deployed VCMs in these areas to battle the myths and misconceptions about vaccines that have led to poor immunization uptake in these areas. With the support of the CGPP and other polio partners, a total of 4.5 million children younger than 5 years were immunized in 2018 alone.^15^ In Yobe state, the CGPP is well recognized by the state Polio Emergency Operations Center and has made important contributions to improving the coverage of various vaccines by reaching children in difficult, hard-to-reach areas of the state. The CGPP has supported the National Polio Eradication Program in Nigeria to address longstanding misconceptions and myths that have resulted in immunization refusal or total immunization rejection. The CGPP has leveraged strong community connections and partnerships to generate positive results.

A critical area of CGPP expertise is the development of context-specific messages in the local language that resonate with families. The solid knowledge of VCMs about polio, child health, and community needs along with the strong relationships that VCMs have with families help to make VCMs effective in community mobilization. Volunteer community mobilizers have raised awareness, dispelled myths, made referrals, and converted parents into acceptors of polio immunization. Furthermore, VCMs provide important information to mothers and caregivers concerning routine immunization, antenatal care, nutrition, and child health. Volunteer community mobilizers in each settlement of the CGPP implementation areas also regularly track the number of new births in their settlements and attend naming ceremonies (*suna*), where they administer OPV to the newborn. Local government area Coordinators and volunteer ward supervisors report their data weekly. The CGPP also provide supportive supervision on how to conduct activities and on-the-job mentorship to improve the efficiency and effectiveness of activities and reporting.

## CONCLUSION

Nigeria is the last polio endemic country in the African region of the WHO. The polio eradication initiative in the country’s North East continues to be challenged by cases of immunization resistance rooted in myths and misconceptions. The CGPP in Nigeria contributed to overcoming resistance to polio eradication activities. The CGPP has evolved several innovative approaches to address high rates of non-compliance and immunization rejection in North East Nigeria, particularly in Yobe state. The CGPP has gained the access and trust of these communities by implementing strategies that contribute to stopping rumors before they start and quelling rumors rapidly once they begin to take shape. These achievements have been a key part of Nigeria’s overall progress in polio eradication since 2014. Similar strategies hold great promise for addressing other public health challenges in Nigeria and beyond, particularly for mothers and children.

## Footnotes

† Circulating vaccine-derived Type 2 poliovirus occurs when the live weakened in OPV genetically changes, circulates, and causes polio. Cases are rare and can occur in populations with under-immunized children. However, cVDPV has been circulating for many years in Nigeria.

‡ The NEOC is the government body charged with the mandate of overseeing Nigeria’s polio eradication effort. It consists of government and implementing partners such as the CGPP, WHO, CDC, UNICEF, Rotary, and the Bill & Melinda Gates Foundation.

§ “Follow the virus” means conducting immunization responses (such as polio campaigns and outbreak responses) by focusing on populations located in areas where the virus has been detected in children younger than 5 years.

‖ Three doses of pentavalent vaccine provide protection against diphtheria, pertussis, tetanus, hepatitis B, and Haemophilus influenzae type B.

¶ Iftar is the Muslim “breaking of the fast” that occurs at dusk during Ramadan. Ramadan is the Islamic month of fasting, which is practiced from sunrise to sunset.

# Ramadan is the ninth month of the Islamic calendar during which time Muslims fast during daylight hours to commemorate the revelation of the Quran to Muhammad.

†† Imams are Muslim religious leaders who lead prayers in mosques.

‡‡ Two cases of AFP from non-polio causes among 100,000 children younger than 15 years are normally expected in a 12-month period.

§§ Community informants conduct passive surveillance. Traditional birth attendants, bone setters, traditional healers, spiritual leaders, and barbers act as informants.

‖‖ The community case definition used by community informants and VCMs are three-prong: children who are younger than 15 years and have sudden weakness in an arm or leg and the limb is floppy.

¶¶ The CGPP team consists of a volunteer community mobilizer, a volunteer ward supervisor, and an LGA Coordinator.

## One community leader remarked: “*Ba wanda yataba zuwa ya wayar mana da kai kamar yanda kukayi saboda haka a madadin mutanen garin zamu cigabada karban alluran rigakafi a koyaushe idan mutanan sukazo*,” meaning “no other person tried to explain these issues patiently before now, but now we will allow our children to be vaccinated.”
